# Exposure to ZnO/TiO_2_ Nanoparticles Affects Health Outcomes in Cosmetics Salesclerks

**DOI:** 10.3390/ijerph17176088

**Published:** 2020-08-21

**Authors:** Ching-Chang Lee, Yi-Hsin Lin, Wen-Che Hou, Meng-Han Li, Jung-Wei Chang

**Affiliations:** 1 Department of Environmental and Occupational Health, College of Medicine, National Cheng Kung University, Tainan 701, Taiwan; cclee@mail.ncku.edu.tw (C.-C.L.); yihsin19940805@gmail.com (Y.-H.L.); 2 Research Center for Environmental Trace Toxic Substances, National Cheng Kung University, Tainan 701, Taiwan; 3 Department of Environmental Engineering, National Cheng Kung University, Tainan 701, Taiwan; whou@mail.ncku.edu.tw (W.-C.H.); amiable0330@gmail.com (M.-H.L.); 4 Institute of Environmental and Occupational Health Sciences, School of Medicine, National Yang-Ming University, Taipei 112, Taiwan

**Keywords:** nanocosmetics, zinc oxide, titanium dioxide, oxidative stress markers

## Abstract

Concerns about the effects of nanoparticles (NPs) on human health are being raised by researchers because the risks of nanocosmetics like sunscreen are unknown. We explored the association between urinary oxidative stress markers and exposure of cosmetics salesclerks to 20 cosmetics that might contain titanium dioxide (TiO_2_)/zinc oxide (ZnO) NPs. We then recruited 40 cosmetics salesclerks and 24 clothing salesclerks and categorized them based on their exposure to ZnO and TiO_2_ NPs. Nineteen and 15 samples met the EU definition for TiO_2_ and ZnO nanomaterials, respectively. Participants with a higher co-exposure index of ZnO and TiO_2_ NPs had a significantly higher base level of urinary 8-hydroxy-2′-deoxyguanosin (8-OHdG) concentrations than the lower co-exposure group (5.82 vs. 2.85 ng/mL, *p* < 0.001). After potential confounding factors had been adjusted for, the TiO_2_ and ZnO NP co-exposure index was significantly positively associated with the urinary 8-OHdG base concentration (β = 0.308, 95% CI = 0.106 to 0.510) and the creatinine-adjusted concentration (β = 0.486, 95% CI = 0.017 to 0.954). Current evidence suggests that the likelihood of harm from using sunscreens containing nanoparticles might result in higher urinary 8-OHdG. However, our limited number and types of sample cosmetics might underestimate the risk.

## 1. Introduction

Recently, consumer products such as cosmetics, clothes, packaging, toys, and even food products created using nanotechnology have been gradually incorporated into our daily life and the list is growing fast [1]. The most common cosmetic that uses nanotechnology (nanocosmetic) is sunscreen. However, studies [2,3] have raised health concerns about nanoparticles (NPs) because their health risks are unknown. Because they are small, nanoparticles enable the skin to easily absorb nanocosmetics; thus, nanomaterials are widely used in skin care products and cosmetics. Most nanocosmetics contain titanium dioxide (TiO_2_) and zinc oxide (ZnO) NPs about 50–100 nm, which are better for ultraviolet (UV) light shielding and transparency than are larger particles.

Concerns relating to the use of nanoparticles are two-fold. First, NPs in nanocosmetics produce more free radicals when exposed to UV light. Although healthy or psoriatic skin absorbs less TiO_2_ or ZnO [4], in real-life situations, uptake through skin cracks, minor injuries, and flexed skin remains to be evaluated. In addition, TiO_2_ NPs sometimes penetrate the skin and might become a major concern for people with healthy skin. Many studies [5,6,7] have reported that exposure to TiO_2_ or ZnO NPs might cause cell death and allergic reactions and might significantly raise urinary 8-hydroxy-2′-deoxyguanosin (8-OHdG) levels and concentrations of inflammatory markers like IL-6, IL-8, and TNF-α. Several studies have suggested that ROS generation and consequent oxidative stress are frequently observed with NP toxicity [8,9]. Oxidative stress reflects a disturbance in the balance between the production and accumulation of reactive oxygen species (ROS) [10]. When ROS concentration is in excess, oxidative damage to proteins, lipids, and DNA occurs, thus causing structural and functional cellular changes. Measuring urinary 8-hydroxy-2-deoxyguanosine (8-OHdG) has some advantages as it is very stable in urine [11], is noninvasive, and its excretion is likely to reflect the oxidative DNA damage [12]. Concentrations of urinary oxidative stress biomarkers have been proposed as an effect biomarker to survey populations exposed to engineered nanomaterials [13,14].

Moreover, TiO_2_ and ZnO NPs absorb significant UV radiation, which in aqueous media, produces hydroxyl species. These species might substantially damage DNA [15,16], therefore raising health concerns about sunscreens [17].

According to one commercial survey [18], the nano-ingredients of nanocosmetics are unclearly labeled because of the strict regulations and complicated certification system. Thus, consumers are liable to expose themselves to NPs without realizing it. Critical information such as nanoparticle size and concentrations are not disclosed for most consumer products; therefore, the actual health hazard of these products remains largely unknown.

There is always conflicting evidence as to whether nanoparticles are small enough to penetrate the epidermis and be absorbed into the human blood stream, causing toxicity with long-term use [19]. Although, some studies have found increasing amounts of zinc in the blood and urine of human trial participants after a five-day application of sunscreen containing ZnO nanoparticles. Besides, the concentration excreted peaked at nine days post-application [20]. Besides, James et al. has also integrated findings from several studies published between 2006 and 2010, which showed that a small amount of Zn (either as nanoparticles or free ions) is able to penetrate healthy human skin and enter the circulation, stimulating an immune response [21]. Indeed, several studies have raised concerns regarding the potential photo-carcinogenicity of nanoparticles in sunscreens [22]. To date, however, there has been inadequate evidence in human models. Cosmetics salesclerks have to recommend cosmetics to customers and they often apply nanocosmetics on their own skin. Therefore, they have a greater chance of experiencing a skin allergy and skin damage, which will increase the nanoparticles exposure. Therefore, the implications for long-term use for this group must be established; thus, we explored the association between urinary oxidative stress markers and exposure to TiO_2_ and ZnO NPs in nanocosmetics salesclerks.

## 2. Materials and Methods

### 2.1. Sunscreen Selection and Analysis

To understand the types and number of consumer products available on the market that have NPs, we used the Nanotechnology Consumer Products Inventory, created by the Woodrow Wilson International Center for Scholars and the Project on Emerging Nanotechnologies, and market research studies and made sure that the products were available in Taiwan [23]. We then purchased cosmetics that we thought would contain TiO_2_ or ZnO NPs and we used single-particle inductively coupled plasma-mass spectrometry (sp-ICP-MS) to analyze the content, concentration, and size of the NPs.

### 2.2. Sample Preparation for sp-ICP-MS Analysis

Ionic Ti and Zn ICP-MS standards, TiO_2_ (P_25_) and ZnO nanopowder (<100 nm/particle), were purchased from Sigma-Aldrich (St. Louis, MO, USA). Nitric acid (HNO_3_) (67–70% [*w*/*w*], Ultrex II) was purchased from J. T. Baker (Phillipsburg, NJ, USA). Ninety-seven percent [*w*/*w*] sulphuric acid (H_2_SO_4_) and 30% [*w*/*w*] hydrogen peroxide (H_2_O_2_) were purchased from Honeywell (Minato City, Japan). Citrate-coated 50-nm gold nanoparticles (AuNPs) were purchased from nanoComposix Inc. (San Diego, CA, USA). All other chemicals were purchased from Sigma-Aldrich or J. T. Baker. All aqueous samples were prepared with water purified by a Millipore Synergy ultrapure water system (≥18.0 MΩ) (Merck Ltd., Taipei, Taiwan). After all the samples had been homogenized, 0.2 g or more of the sunscreen sample was dispersed in a 1% Triton X-100 aqueous solution to make a 0.1% (*v*/*v*) suspension. Here, an aliquot of 50 mg product lotion was then added to a 1% (*v*/*v*) Triton X-100 aqueous solution (50 mL in centrifuge tubes), resulting in a 0.1% (*w*/*v*) suspension. The mixture was sonicated and vortexed until no aggregates could be seen.

### 2.3. sp-ICP-MS Method

The typical instrument condition and settings used in sp-ICP-MS analysis are given in Table S1. Aqueous samples were nebulized using a concentric nebulizer in a cyclone spray chamber and ionized using argon plasma. The sample flow rate (range: 0.28–0.36 mL/min) was determined daily. Particle size quantification was based on a calibration curve constructed from a 2% HNO_3_ blank and five concentration levels of dissolved Ti and Zn standard solutions (range: 0.1 to 10 μg/L) [24]. To measure transport efficiency (η), 50 nm standard gold nanoparticles (AuNPs) (at ~10^5^ particles/mL) was spiked into the samples as described by Pace et al. The transport efficiencies were in the range of 4.5–7.0% during this study.

The signal intensity (INP) obtained from sample analysis was converted to the diameter by estimating the size of the spherical TiO_2_ particle using Equation (1):

(1) $$d\;=\;{\left(\frac{6\;\times \;I_{NP}}{R\;\times \;f_{a}\;\times \;\rho \;\times \;\pi }\right)}^{1/3},$$

where *d* is the diameter; *R*, which defines the detector sensitivity of the instrument, is determined from the ionic standards; *f_a_* is the mass fraction of the metal element in the chemical formula of an NP; and *ρ* is the mass density. Size distribution can then be obtained by plotting *d* against the frequency of NP occurrence. NP concentration (N_p_) was determined using Equation (2):

(2) $$N_{p}\;=\;\frac{f_{p}}{q\;\times \;t_{s}\;\times \;\eta }$$

where *f_p_* is the number of particles resolved per analysis; *q* is the sample flow rate; *t_s_* is the scan time per analysis; and η is the transport efficiency. The sp-ICP-MS method used in this study can detect NPs in the concentration range of 1000–100,000 particles/mL with quantitative recovery and good sizing capability in complex matrices such as wastewater, based on our previous results (not shown).

For each sample, the isotopes ^47^Ti and ^64^Zn were detected using 0.1 ms dwell time for a total measurement time of 60 s (=600,000 data points).

After each run, software (Syngistix Nano Application Module 1; PerkinElmer, Taipei, Taiwan) automatically integrated the peak area of each single particle and generated information about particle size distribution, particle concentration, and dissolved concentration.

### 2.4. Recruiting Participants

We recruited full-time cosmetics salesclerks in retail stores or cosmetics product distributors in department stores for the high-exposure group and clothing salesclerks in the same department stores in southern Taiwan for the control group. We first excluded recruits who had immune dysfunction or who frequently used general nanotechnology-based consumer products. We also excluded exposure group recruits who did not demonstrate products for customers. The Human Ethics Committee of National Cheng Kung University Hospital approved the study protocol (#: B-ER-105-416). All participants provided written informed consent. Finally, the exposure group contained 40 salesclerks and the control group contained 24.

### 2.5. Sample Collection

All glassware was washed with organic solvents and packed with aluminum foil before we collected samples. From 2 January 2017 to 8 May 2017, urine samples were collected from each participant on four separate occasions. On each occasion, we collected two urine samples (20–30 mL), one pre-shift (first morning spot urine) on Thursday and another post-shift on Sunday. We conducted interviews to determine exposure scenarios and filled in standardized nanomaterials exposure questionnaires to obtain demographic characteristics.

### 2.6. Daily Exposure Dose and Cumulative Risk Calculation

Chronic exposure is continuous or repeated contact with a toxic substance over a long period of time (months or years). For the cosmetics which contain TiO_2_ and ZnO NPs that are used every day by these cosmetics salesclerks, the exposure would be chronic. Therefore, we have established an exposure index to evaluate the long-term effect of NPs exposure, which integrated the nanoparticle concentration of the cosmetics and the usage of nano-consumer products to evaluate the relationship between oxidative stress and chronic TiO_2_ or ZnO NP exposure, which is shown in Equation (3):

(3) $$\mathrm{Exposure}\;\mathrm{Index}\;(\mathrm{particles}\;{\mathrm{kg}}^{-1}\;{\mathrm{day}}^{-1})\;=\;\frac{C\;\times \;\mathrm{Exp}.\;D}{\mathrm{BW}}\times \frac{W\_\mathrm{hr}}{24}\times \frac{W\_\mathrm{day}}{365}\times \frac{\mathrm{ED}}{\mathrm{AT}},$$

where C (particles/g) is the concentration of sunscreen; Exp. D is the exposure dose, which was calculated from the frequency of using sunscreen and the number of salesclerks; Working hours (W_h), working days (W_day), seniority (ED), and body weight (BW) were collected from the questionnaire. Life span (AT) was taken from exposure-factor surveys [25].

### 2.7. Determining 8-Hydroxy-2′-Deoxyguanosine (8-OHdG)

A competitive enzyme-linked immunosorbent assay (ELISA) (BIOXYTECH^®^ 8-OHdG-EIA™ kit; OXIS Health Products Inc., Portland, OR, USA) was used to quantitatively measure oxidative DNA damage in urine samples. Briefly, after the necessary treatment, cellular DNA was isolated using a DNA extraction kit (iNtRON Biotechnology Inc., Sungnam, Korea). The quantity of 8-OHdG, a deoxyriboside form of 8-oxoguanine in the DNA, was determined using a microplate reader on a standard curve measured at 450 nm absorbance.

### 2.8. Statistical Analysis

We used descriptive statistics to describe the distributions of NP demographic data and NP size of TiO_2_ and ZnO. The participants were categorized into two groups based on their professions. The Kruskal-Wallis test was used to assess differences in oxidative stress levels and demographic characteristics between the two groups. We used a χ^2^ test to determine the differences in demographic characteristics between the two groups. We also used a multivariate linear regression analysis to evaluate the association between oxidative stress levels and exposure to TiO_2_ and ZnO.

SPSS 22 (IBM Corp., Armonk, NY, USA) was used for all statistical analyses. Significance was set at *p* < 0.05.

## 3. Results

### 3.1. Size and Concentration of TiO_2_ and ZnO NPs in Sunscreens

After we completed our market survey of cosmetic products containing ZnO or TiO_2_, we bought 20 products (all milky or cream cosmetics with UV protection) to analyze the NPs. The SP ICP-MS geometric mean diameter (nm), particle number concentration (particles/g), and metal oxide content (% weight) are reported in Table 1. TiO_2_ NPs were detected in 19 sunscreens, with geometric mean diameter (Dg) values from 71.4 to 112 nm; ZnO NPs were detected in all sunscreens, with Dg values from 57.7 to 144.4 nm. The TiO_2_ weight content in sunscreens ranged from 1% to 30.7% and that of ZnO from 1% to 9.14%. Many ZnO NPs were detected in six sunscreens (#002, #005, #007, #011, #013, and #020), with Dg values from 98.2 to 144.4 nm; few ZnO NPs (9.14% at maximum) were detected in all sunscreens (Table 2).

Although the analysis results showed that all the products contained ZnO and TiO_2_, ZnO and TiO_2_ were not labeled as ingredients on some products. The measurement of TiO_2_ indicates that 19 samples met the EU definition for nanomaterials (>50% by number of particles with a size <100 nm) and the mode sizes ranged from 52 to 68 nm. In another analysis, 15 products met the EU definition for nanomaterials in ZnO: mode sizes ranged from 48 to 83 nm.

### 3.2. Study Population

We recruited 40 department store cosmetics salesclerks and 24 clothing salesclerks. The average age of the clothing salesclerks was higher than that of the cosmetics salesclerks (42.2 vs. 27.3 years, *p* < 0.001) (Table 3). Moreover, the clothing salesclerks had a significantly higher average BMI than the cosmetics salesclerks did (23.2 vs. 20.7 kg/m^2^, *p* < 0.001).

Moreover, the cosmetics salesclerks had higher education and economic levels than the clothing salesclerks did. Cosmetics salesclerks had more weekday working time than the clothing salesclerks did.

Cosmetics salesclerks used more cosmetics (lotions, lipstick, powder foundation, and eye shadow) than the clothing salesclerks did (Table S2).

### 3.3. Urinary 8-OHdG Analysis in Nanocosmetics Salesclerks

All salesclerks were first categorized based on their job title. Baseline 8-OHdG concentrations in the cosmetics salesclerks were significantly higher than those in the clothing salesclerks (5.42 vs. 2.53 ng/mL, *p* = 0.001) in Thursday pre-shift urine, but there were no significant differences between the Sunday post-shift concentrations, not even after creatinine had been adjusted for. We excluded salesclerks with abnormal creatinine (<30 or >300 mg/dL) levels. Baseline 8-OHdG concentrations in the cosmetics salesclerks were significantly higher than in the clothing salesclerks in both Thursday pre-shift and Sunday post-shift urine (5.41 vs. 2.85 ng/mL, *p* = 0.004; 4.90 vs. 2.85 ng/mL, *p* = 0.037) (Table S3).

**Figure 1:**
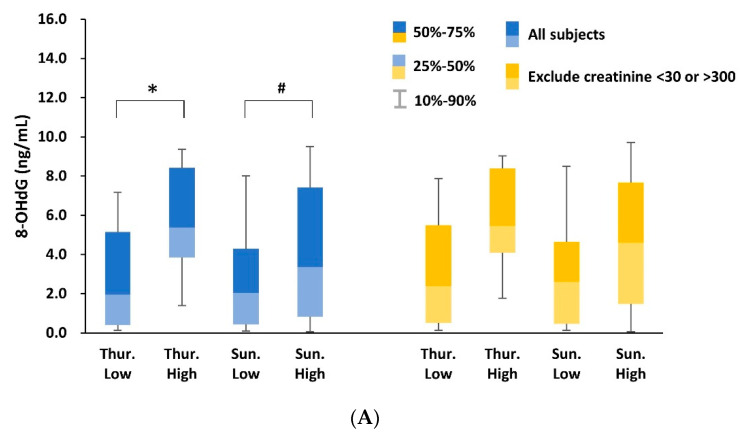

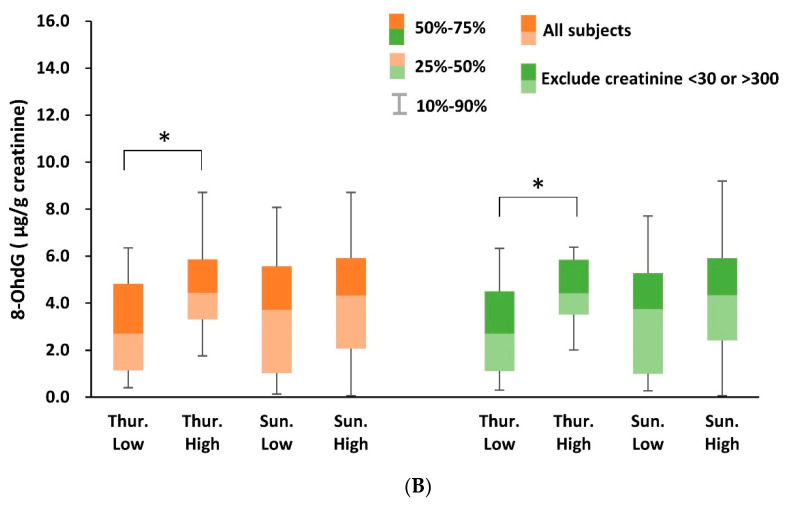
Oxidative stress analytical results grouped by Co-Exposure index integrating ZnO and TiO_2_ NPs showing in (**A**) ng/mL and (**B**) μg/g creatinine. ^#^ *p* < 0.1; * *p* < 0.05

All salesclerks were then categorized based on the co-exposure index of ZnO and TiO_2_ NPs (Figure 1 and Table S4). Baseline 8-OHdG concentrations in the high-exposure group were significantly higher than in the low-exposure group (5.82 and 2.85 ng/mL, *p* = 0.001).

The exposure indices of TiO_2_ NPs were significantly positively associated with baseline and creatinine-adjusted 8-OHdG concentrations (β = 0.417, *p* < 0.001, and β = 0.334, *p* < 0.001) (Table S5).

After adjusting for potential confounding factors, the exposure index of TiO_2_ NPs were significantly positively associated with both the urinary 8-OHdG original and creatinine-adjusted concentration (β = 0.383, 95% CI = 0.176 to 0.589, and β = 0.648, 95% CI = 0.167 to 1.131) (Table 4 and Table S6). Moreover, after adjusting for potential confounding factors, the TiO_2_ and ZnO NPs co-exposure indexes were significantly positively associated with both the urinary 8-OHdG original and creatinine-adjusted concentration (β = 0.308, 95% CI = 0.106 to 0.510, and β = 0.486, 95% CI = 0.017 to 0.954) (Table 4 and Table S6).

## 4. Discussion

### 4.1. Findings

Our most important finding was that all the products we bought and analyzed contained ZnO and TiO_2_; however, some of the products did not include ZnO and TiO_2_ as ingredients on their labels. Nineteen TiO_2_ samples met the EU definition for nanomaterials (>50% <100 nm by number of particles/g); mode sizes ranged from 52 to 68 nm. Another analysis showed that 15 ZnO samples met the EU definition for ZnO nanomaterials. Smijs and Pavel (2011) reported that TiO_2_ was more effective in the UVB range and ZnO in the UVA range [26]. Broadband UV protection might be more significant than these two chemicals combined. We also found that ZnO and TiO_2_ NPs had been measured in commercial sunscreens [27,28].

The significantly positive correlation between urinary 8-OHdG levels and the exposure index of TiO_2_ NPs and the co-exposure index integrating TiO_2_ and ZnO NPs indicated that exposure to NPs might affect the oxidative stress of salesclerks despite inconsistent evidence that NPs are small enough to penetrate the epidermis and the blood stream [19]. Graille et al. established a standardized protocol for a systematic review and meta-analysis to assess background ranges for urinary 8-OHdG concentrations in healthy populations. He observed a significant positive association between smoking status and urinary 8-OHdG concentrations when measured by chemical analysis [29]. Besides, plasma 8-OHdG was found to be positively associated with age, suggesting the accumulation of oxidative damage with increasing age. This finding supports the hypothesis that oxidative damage accumulates with time and so contributes to ageing and age-related disease [30]. Mizoue et al. has reported an inverse association between BMI and urinary 8-OHdG levels using an intervention study [31] as well as other cross-sectional studies [32,33]. Moreover, a modifying effect of smoking on their relationship was also suggested [31,32]. Besides, urinary excretion of 8-OHdG is associated with smoking, a significant carcinogenic factor [31,32,33]. A pilot investigation also showed that leanness was associated with higher levels of aromatic DNA adducts among smokers [34]. Moreover, a modifying effect of smoking on their relationship was also suggested [31,32]. Besides, urinary excretion of 8-OHdG is associated with smoking in two cross-sectional studies [31,32,33].

Most TiO_2_-containing sunscreens will lead to the photo-oxidation of phenol and damage human DNA using TiO_2_ as a catalyst under sunlight [15]. This is in contrast to Gulson et al. (2010), which reported more ZnO in the blood and urine of human volunteers after a five-day continuous application of ZnO NPs-containing sunscreen [18]. Moreover, James et al., 2013 claimed that a small amount of Zn (either as NPs or free ions) can penetrate healthy human skin, enter the circulation, and stimulate an immune response [19]; they concluded that macrophages are important for protecting the body against the cytotoxic effects of ZnO NPs.

Martorano et al. (2010) reported that UVB irradiation causes Zn^2+^ dissociation in ZnO sunscreen and therefore, the accumulation of imbalanced Zn^2+^ causes cytotoxicity and oxidative stress [35]. ZnO or TiO_2_ NPs can produce a series of biochemical and cellular signaling events—ROS induction, DNA damage, and cell death mediated by activating NF-κB, a transcription factor of proinflammatory responses [36], proinflammatory cytokine secretion [37], and endoplasmic reticulum stress induction [38]. Of great importance to the potential adverse effects of NPs in sunscreens, TiO_2_ and ZnO NPs have been found with cytotoxicity in different human skin models, principally HaCa T cells (immortalized human keratinocytes), human or animal-derived skin samples, and human volunteers [39]. When HaCa T cells were incubated with 10 to 500 μg mL^−1^ of TiO_2_ NPs for 2 h, a significant reduction in cell viability and induction of ROS also occurred [40]. After daily oral administration of TiO_2_ for 6 months, lower body weight and significant kidney pathology were found in mice and could possibly be attributable to NP-induced oxidative stress [41]. TiO_2_ NPs caused liver injury and induced oxidative stress and DNA damage in hepatocytes of mice when administered for 2 weeks at doses >100 mg/kg [42].

### 4.2. Limitations

This study has limitations. High exposure levels to TiO_2_ and ZnO NPs might raise 8-OHdG concentrations. We analyzed only liquid and milky products, but even TiO_2_ and ZnO NPs might be added to other solid, cream, or spray-type cosmetics as well; therefore, the risk might be underestimated. Hence, more product categories must be included to conduct further simultaneous exposure assessments. The benefits of sunscreens reduce skin cancer risk, which far outweighs the potential risks of long-term use. Nano-sized TiO_2_ might also be widely used in toothpastes [43,44].

## 5. Conclusions

Oxidative DNA damage is a major event during NP-induced injury that needs to be thoroughly characterized in order to predict NP-induced toxicity. Some diseases, such as cardiovascular or chronic obstructive pulmonary diseases (COPD), have also been associated with excessive concentrations of 8-OHdG [45,46]. In the present study, we have observed that the likelihood of harm from using sunscreens containing nanoparticles might result in higher urinary 8-OHdG. Sunscreens reduce skin cancer risk, which far outweighs the potential risks of long-term use. However, the exposure risk via using certain cosmetics cannot be ignored. Further research is required to support our findings.

## Figures and Tables

**Table 1:** Size, number, and weight concentration of TiO_2_ in sunscreens by SP-ICP-MS.

Sample ID	SPF	Geo-Mean Size (nm)	TiO_2_ # Concentration (#/g Product)	Mode Size (nm)	Fraction of Nanosized TiO_2_^1^ (%)	TiO_2_ Weight Percentage ^2^ (%)	TiO_2_ Labeling
#001	50+	98.8	6.04 × 10^9^	55.6	64	0.01	No
#002	30	85.2	1.13 × 10^13^	62.3	77	3.17	Yes
#003	40	75.1	2.00 × 10^13^	60.3	91	2.40	Yes
#004	0	268.3	4.91 × 10^12^	111.0	0	30.07	Yes
#005	50+	91.8	1.50 × 10^12^	61.3	66	0.62	Yes
#006	15	72.8	1.02 × 10^12^	53.7	86	0.16	Yes (1.23%)
#007	50+	71.8	1.25 × 10^11^	52.7	84	0.03	No
#008	50	74.6	7.86 × 10^11^	58.0	85	0.10	Yes
#009	50	75.6	1.06 × 10^11^	52.8	80	0.03	Yes
#010	19	71.4	2.23 × 10^12^	54.0	89	0.55	Yes
#011	50+	86.9	2.15 × 10^11^	65.6	78	0.06	Yes
#012	50	81.8	7.00 × 10^12^	66.0	85	1.23	Yes
#013	50+	101.5	1.83 × 10^7^	66.9	65	0.00	No
#014	50	79.6	7.34 × 10^12^	63.2	85	1.23	Yes
#015	15	107.8	2.54 × 10^12^	63.7	57	2.84	Yes
#016	50	94.1	8.39 × 10^12^	56.6	61	3.06	Yes
#017	24	82.2	1.76 × 10^12^	62.6	80	0.37	Yes
#018	50+	106.4	3.60 × 10^10^	64.1	54	0.03	No
#019	20	74.3	5.09 × 10^12^	56.1	87	1.21	Yes
#020	25	112.0	1.14 × 10^12^	68.0	50	1.24	Yes

#: number of particles; ^1^: The ratio of the number concentration of nano-size TiO_2_ and full-range particle size TiO_2_; ^2^: The ratio of the mass concentration of nano-size TiO_2_ and full-range particle size TiO_2_.

**Table 2:** Size, number, and weight concentration of ZnO in sunscreens by SP-ICP-MS.

Sample ID	SPF	Geo-Mean Size (Nm)	Zno # Concentration (#/G Product)	Mode Size (Nm)	Fraction of Nanosized Zno ^1^ (%)	Zno Weight Percentage ^2^ (%)	Zno Labelled Labeling
#001	50+	57.7	3.66 × 10^7^	51.6	95	0.00	No
#002	30	102.3	4.51 × 10^11^	83.0	53	0.22	No
#003	40	66.2	2.22 × 10^7^	67.1	94	0.00	No
#004	0	61.7	1.64 × 10^8^	62.4	100	0.00	No
#005	50+	98.2	2.36 × 10^12^	72.1	30	1.72	Yes (12.53%)
#006	15	69.7	4.29 × 10^7^	59.4	88	0.00	No
#007	50+	136.0	9.19 × 10^12^	91.5	21	9.14	Yes (9.45%)
#008	50	55.6	1.72 × 10^8^	48.5	98	0.00	No
#009	50	67.0	1.37 × 10^7^	50.2	89	0.00	No
#010	19	65.2	5.42 × 10^7^	55.3	93	0.00	No
#011	50+	101.0	6.16 × 10^11^	96.1	39	0.37	Yes (7.36%)
#012	50	99.1	1.27 × 10^7^	80.5	60	0.00	No
#013	50+	144.4	6.98 × 10^11^	103.3	16	1.22	Yes (9.59%)
#014	50	92.2	1.68 × 10^8^	59.0	69	0.00	No
#015	15	67.7	1.51 × 10^9^	57.3	97	0.00	No
#016	50	88.7	1.51 × 10^8^	73.4	79	0.00	No
#017	24	96.0	1.48 × 10^8^	78.9	74	0.00	No
#018	50+	91.6	3.02 × 10^6^	60.2	62	0.00	No
#019	20	77.7	1.32 × 10^8^	72.4	86	0.00	No
#020	25	106.3	4.34 × 10^12^	76.4	42	2.47	No

#: number of particles; ^1^: The ratio of the number concentration between nano-size ZnO and full-range particle size ZnO; ^2^: The ratio of the mass concentration between nano-size ZnO and full-range particle size ZnO.

**Table 3:** Demographic characteristics of the study population grouped by sampling site.

	Cosmetics Salesclerks (*n* = 40)	Clothing Salesclerks (*n* = 24)	*p* ^a,b^
Age (years) ^c^	27.3 (20–47)	42.2 (23–54)	<0.001
Weight (kg) ^d^	54.4 (6.56)	60.6 (10.1)	0.004
Height (cm) ^d^	161.8 (5.27)	161.5 (6.62)	0.812
BMI	20.7 (2.0)	23.2 (3.28)	<0.001
Marital status ^e^			
Unmarried/Divorced	29 (72.5)	11 (45.9)	0.033
Married	11 (27.5)	13 (54.2)	
Educational level			
≤High school	15 (37.5)	17 (70.9)	0.069
Tech school	4 (10.0)	2 (8.3)	
Bachelor’s degree	21 (52.5)	5 (20.8)	
Monthly income (NT$)			
<24,999	11 (27.5)	9 (37.5)	0.048
25,000~29,999	5 (12.5)	9 (37.5)	
30,000~34,999	15 (37.5)	5 (20.8)	
35,000~39,999	5 (12.5)	1 (4.2)	
40,000~44,999	4 (10.0)	0 (0.0)	
Seniority (years)	4.72 (5.70)	11.9 (7.70)	<0.001
Working time (day/month)	20.5 (2.80)	21.3 (2.63)	0.262
Weekday working time (h/day)	8.59 (0.53)	7.96 (2.13)	0.078
Weekend working time (h/day)	8.70 (0.71)	8.52 (2.03)	0.611
Exposed to Tobacco Smoke Exposure			
Active	7 (17.5)	2 (8.3)	0.264
passive	13 (32.5)	10 (41.7)	0.317
Alcohol drinker ^f^	5 (12.5)	0 (0.0)	0.086
Tea drinker ^f^	26 (65.0)	13 (54.2)	0.275
Coffee drinker ^f^	15 (37.5)	14 (58.3)	0.087

^a^ Continuous variables between two groups were compared using the Kruskal-Wallis test; ^b^ Categorical variables were compared with χ^2^ test; ^c^ Expressed as Mean (range); ^d^ Expressed as Mean ± SD; ^e^ n (%); ^f^ Once a week at least.

**Table 4:** Multiple regression analysis of urinary 8-OHdG concentration and exposure index ^a^.

Exposure Index ^b^	ZnO NPs	TiO_2_ NPs	ZnO and TiO_2_ NPs
Urinary 8-OHdG (ng/mL) ^c^	β = −0.047 (−0.272, 0.177)	β = 0.383 ** (0.176, 0.589)	β = 0.308 ** (0.106, 0.510)
Urinary 8-OHdG (μg/g creatinine) ^c^	β = −0.087 (−0.600, 0.426)	β = 0.649 ** (0.167, 1.131)	β = 0.486 * (0.017, 0.954)

^a^ Adjusted for BMI, smoking, alcohol drinking, and tea drinking habits; ^b^ log-transformations of exposure index was used; ^c^ Expressed as coefficient β and 95% CI; * *p* < 0.05; ** *p* < 0.01.

## Supplementary Materials

The following are available online at https://www.mdpi.com/1660-4601/17/17/6088/s1, Table S1: SP-ICP-MS analytical conditions, Table S2: Personal use of cosmetics grouped by commodity classification Table S3: Oxidative stress analytical results grouped by commodity classification, Table S4: Oxidative stress analytical results grouped by Co-Exposure index integrating ZnO and TiO2 NPs, Table S5: The association between urinary 8-OHdG concentration and exposure index ^a^, Table S6A: Multiple regression analysis of urinary 8-OHdG concentration (ng/mL) and ZnO NPs exposure ^a^, Table S6B: Multiple regression analysis of urinary 8-OHdG concentration (μg/g creatinine) and ZnO NPs exposure ^a^, Table S6C: Multiple regression analysis of urinary 8-OHdG concentration (ng/mL) and TiO2 NPs exposure ^a^, Table S6D: Multiple regression analysis of urinary 8-OHdG concentration (μg/g creatinine) and TiO2 NPs exposure ^a^, Table S6E: Multiple regression analysis of urinary 8-OHdG concentration (ng/mL) and ZnO and TiO2 NPs exposure ^a^, Table S6F: Multiple regression analysis of urinary 8-OHdG concentration (μg/g creatinine) and ZnO and TiO2 NPs exposure ^a^.

## Abbreviations

NPs	nanoparticles
TiO_2_	titanium dioxide
ZnO	zinc oxide
8-OHdG	8-hydroxy-2′-deoxyguanosin
UV	ultraviolet
sp-ICP-MS	single-particle inductively coupled plasma-mass spectrometry
ELISA	enzyme-linked immunosorbent assay
