# Supplementary material for: Exposure to ZnO/TiO2 Nanoparticles Affects Health Outcomes in Cosmetics Salesclerks
Source: Int J Environ Res Public Health. 2020 Aug 21;17(17):6088. doi: 10.3390/ijerph17176088 (PMC7504197; doi:10.3390/ijerph17176088)
Supplement: Supplementary file 1 [file ijerph-17-06088-s001.pdf]

Table S1. SP-ICP-MS analytical conditions.

Parameter	Values
Sample flow rate, q (mL/min)	0.28-0.36
Dwell time (ms)	Nano mode: 0.1
RPq	0.5
RF power (W)	1650
Scan time, ts (s)	60
Data acquisition mode	Nano mode: Time-resolved analysis
Transport efficiency, η (%)	4.5-7.0
Mass monitored	^{47}Ti, ^{64}Zn

Table S2. Personal use of cosmetics grouped by commodity classification.

Cosmetics	Cosmetic sales (n=40)	Clothing sales (n=24)	P-value [a]
Toning lotion (cm^3/week) [b, c]	13.4 (4.82)	9.28 (5.27)	0.002**
Face lotion (cm^3/week)	7.31 (6.16)	4.34 (4.20)	0.041*
Day cream (cm^3/week)	5.03 (5.90)	4.02 (5.52)	0.498
Night cream (cm^3/week)	1.16 (1.98)	0.54 (1.23)	0.168
Liquid foundation (cm^3/week)	4.94 (2.47)	2.24 (2.25)	<0.001**
Body lotion (cm^3/week)	20.5 (18.6)	25.7 (17.8)	0.278
Body wash (cm^3/week)	32.8 (10.2)	19.0 (17.7)	<0.001**
Shampoo (cm^3/week)	34.6 (10.6)	32.5 (7.57)	0.394
Lipstick (layer/week)	33.2 (22.0)	12.7 (16.4)	<0.001**
Lip balm (layer/week)	18.7 (16.8)	14.0 (13.1)	0.248
Perfume (mL/week)	2.87 (3.02)	0.90 (0.93)	0.003**
Hand nail polish(layer/week)	1.43 (2.81)	0.25 (0.85)	0.051#
Foot nail polish(layer/week)	0.30 (1.02)	0.04 (0.20)	0.225
Blush (layer/week)	10.1 (9.44)	6.29 (8.74)	0.111
Hair gel (layer/week)	2.98 (10.1)	0.88 (3.14)	0.329
Hair spray (mL/week)	0.28 (1.34)	0.00 (0.00)	0.307
Antiperspirant (mL/week)	0.02 (0.05)	0.00 (0.00)	0.122
Powder foundation (cm^3/week)	5.91 (3.50)	1.69 (1.48)	<0.001**
Eye shadow (layer/week)	6.75 (4.17)	1.42 (2.83)	<0.001**

[a] Continuous variables between two groups were compared using Kruskal-Wallis test. [b] Expressed as Mean (SD). [c] Unit/week: Quantify the amount of use × Frequency. # $P<0.1$; * $P<0.05$; ** $P<0.01$.

Table S3. Oxidative stress analytical results grouped by commodity classification.

	Thursday Pre-shift Urine			Sunday Post-shift Urine		
	**Cosmetic sales (n = 40)**	**Clothing sales (n = 24)**	**P-value[a,b]**	**Cosmetic sales (n=40)**	**Clothing sales (n=24)**	**P-value**
8-OHdG (ng/mL)[c]	5.42 (3.45)	2.53 (2.64)	0.001**	4.35 (3.55)	2.62 (3.38)	0.060#
8-OHdG (μg/g creatinine)[c]	6.34 (11.7)	2.91 (2.47)	0.161	4.51 (2.65)	4.11 (7.06)	0.744
Creatinine (mg/dL)[c]	125 (97.5)	95.6 (91.4)	0.234	95.0 (67.3)	79.0 (44.6)	0.303
Protein[d]	18 (45.0)	7 (29.2)	0.161	9 (22.5)	4 (16.7)	0.411
Glucose urine[d]	0 (0.0)	0 (0.0)	-	0 (0.0)	1 (4.2)	0.375
Bilirubin[d]	0 (0.0)	0 (0.0)	-	0 (0.0)	0 (0.0)	-
Urobilinogen[d]	0 (0.0)	0 (0.0)	-	0 (0.0)	0 (0.0)	-
Occult Blood[d]	7 (17.5)	2 (8.3)	0.264	4 (10.0)	3 (12.5)	0.529
	Cosmetics salesclerks (n = 35)	Clothing salesclerks (n = 18)	P-value	Cosmetic sales (n=34)	Clothing sales (n=22)	P-value
8-OHdG (ng/mL)[e]	5.41 (3.06)	2.85 (2.67)	0.004**	4.90 (3.55)	2.85 (3.44)	0.037*
8-OHdG (μg/g creatinine)[e]	4.13 (1.71)	2.93 (2.64)	0.051#	4.45 (2.48)	4.47 (7.27)	0.983
Creatinine (mg/dL)[e]	125 (61.3)	103 (72.7)	0.240	108 (65.3)	87.2 (41.3)	0.207

[a]Continuous variables between two groups were compared using the Kruskal-Wallis test, [b]Categorical variables were compared with Chi-Square test, [c] Expressed as Mean (SD), [d] Abnormal n(%) , [e] Exclude those who with abnormal creatinine (WHO Reference: 30 - 300 mg/dL), #P<0.1, * *P*<0.05; ** *P*<0.01.

Table S4. Oxidative stress analytical results grouped by Co-Exposure index integrating ZnO and TiO_2 NPs.

	Thursday Pre-shift Urine			Sunday Post-shift Urine		
	**High (n = 32)**	**Low (n = 32)**	**P-value[a,b]**	**High (n = 32)**	**Low (n = 32)**	**P-value**
8-OHdG (ng/mL)[c]	5.82 (3.34)	2.85 (2.91)	< 0.001**	4.44 (3.87)	2.91 (3.14)	0.086#
8-OHdG (μg/g creatinine)[c]	6.89 (13.0)	3.22 (2.36)	0.122	4.94 (6.01)	3.78 (3.02)	0.332
Creatinine (mg/dL) [c]	144 (105)	84.2 (75.7)	0.011*	102 (68.1)	75.5 (47.7)	0.072#
Protein [d]	17 (53.1)	8 (25.0)	0.020*	8 (25.0)	5 (15.6)	0.268
Glucose urine [d]	0 (0.0)	0 (0.0)	-	0 (0.0)	1 (3.1)	0.500
Bilirubin [d]	0 (0.0)	0 (0.0)	-	0 (0.0)	0 (0.0)	-
Urobilinogen [d]	0 (0.0)	0 (0.0)	-	0 (0.0)	0 (0.0)	-
Occult Blood [d]	5 (15.6)	4 (12.5)	0.500	3 (9.4)	4 (12.5)	0.500
8-OHdG (ng/mL) [e]	5.90 (2.72)	3.12 (2.98)	0.001**	4.84 (3.84)	3.30 (3.24)	0.112
8-OHdG (μg/g creatinine) [e]	4.41 (1.84)	3.02 (2.20)	0.016*	5.17 (6.19)	3.68 (2.84)	0.258
Creatinine (mg/dL) [e]	147 (61.6)	86.8 (55.5)	<0.001**	114 (65.1)	85.2 (57.8)	0.064#

Exposure to ZnO/TiO_2 NPs leads to higher 8-OHdG

[a] Continuous variables between two groups were compared using Kruskal-Wallis test, [b] Categorical variables were compared with Chi-Square test, [c] Expressed as Mean (SD), [d] Abnormal n (%), [e] Exclude those who with abnormal creatinine (WHO Reference: 30 - 300 mg/dL), [#]P<0.1, * *P*<0.05; ** *P*<0.01.

Table S5. The association between urinary 8-OHdG concentration and exposure index[a].

Exposure Index	Urinary 8-OHdG (ng/mL)	Urinary 8-OHdG (μg/g creatinine)
ZnO NPs [b]	-0.164[#]	-0.037
TiO_2 NPs	0.417**	0.334**
ZnO and TiO_2 NPs	0.304**	0.222*

[a] Spearman correlation analysis; [b] Exposure index value of 0 were replaced by 0.5; [#] P<0.1, * P<0.05, ** P<0.01.

Table S6A. Multiple regression analysis of urinary 8-OHdG concentration (ng/mL) and ZnO NPs exposure[a]

	β	SE	95% CI	P
Intercept	4.526	2.876	-1.168 to 10.219	0.118
BMI	-0.083	0.115	-0.310 to 0.145	0.474
Smoking (Yes)	0.003	1.031	-2.037 to 2.044	0.997
Alcohol drinking (Yes)	3.112	1.325	0.489 to 5.764	0.020
Tea drinking	1.829	0.745	0.355 to 3.304	0.015
ZnO NPs [b]	-0.047	0.114	-0.272 to 0.177	0.677

[a] Adjusted for BMI, smoking, alcohol drinking and tea drinking habits; [b] log-transformations of exposure index was used;.

Table S6B. Multiple regression analysis of urinary 8-OHdG concentration (μg/g creatinine) and ZnO NPs exposure[a].

	β	SE	95% CI	P
Intercept	6.971	6.554	-6.004 to 19.947	0.290
BMI	-0.239	0.262	-0.758 to 0.280	0.363
Smoking (Yes)	3.155	2.349	-1.495 to 7.806	0.182
Alcohol drinking (Yes)	-2.045	3.019	-8.021 to 3.932	0.500
Tea drinking	-0.620	1.698	-3.980 to 2.741	0.716
ZnO NPs [b]	-0.087	0.259	-0.600 to 0.426	0.738

[a] Adjusted for BMI, smoking, alcohol drinking and tea drinking habits; [b] log-transformations of exposure index was used.

Table S6C. Multiple regression analysis of urinary 8-OHdG concentration (ng/mL) and TiO_2 NPs exposure[a]

	β	SE	95% CI	P
Intercept	-0.260	2.758	-5.721 to 5.200	0.925
BMI	0.065	0.109	-0.151 to 0.281	0.551

Smoking (Yes)	-0.036	0.974	-1.963 to 1.892	0.971
Alcohol drinking (Yes)	1.667	1.314	-0.935 to 4.269	0.207
Tea drinking	1.959	0.595	0.780 to 3.138	0.001
TiO_2 NPs [b]	0.383	0.104	0.176 to 0.589	<0.001

[a] Adjusted for BMI, smoking, alcohol drinking and tea drinking habits; [b] log-transformations of exposure index was used.

Table S6D. Multiple regression analysis of urinary 8-OHdG concentration (μg/g creatinine) and TiO_2 NPs exposure[a].

	β	SE	95% CI	P
Intercept	-1.210	6.437	-13.953 to 11.533	0.851
BMI	0.014	0.254	-0.490 to 0.517	0.957
Smoking (Yes)	3.083	2.272	-1.415 to 7.581	0.177
Alcohol drinking (Yes)	-4.485	3.068	-10.558 to 1.587	0.146
Tea drinking	-0.377	1.390	-3.128 to 2.374	0.787
TiO_2 NPs [b]	0.649	0.243	0.167 to 1.131	0.009

[a] Adjusted for BMI, smoking, alcohol drinking and tea drinking habits; [b] log-transformations of exposure index was used.

Table S6E. Multiple regression analysis of urinary 8-OHdG concentration (ng/mL) and ZnO and TiO_2 NPs exposure[a].

	**β**	**SE**	**95% CI**	**P**
Intercept	0.148	2.846	-5.486 to 5.781	0.959
BMI	0.046	0.111	-0.174 to 0.265	0.681
Smoking (Yes)	-0.009	0.990	-1.968 to 1.950	0.993
Alcohol drinking (Yes)	2.106	1.317	-0.502 to 4.714	0.112
Tea drinking	2.137	0.607	0.935 to 3.338	0.001
ZnO and TiO_2 NPs [b]	0.308	0.102	0.106 to 0.510	0.003

[a] Adjusted for BMI, smoking, alcohol drinking and tea drinking habits; [b] log-transformations of exposure index was used.

Exposure to ZnO/TiO_2 NPs leads to higher 8-OHdG

Table S6F. Multiple regression analysis of urinary 8-OHdG concentration (μg/g creatinine) and ZnO and TiO_2 NPs exposure[a]

	β	SE	95% CI	P
Intercept	-0.059	6.609	-13.142 to 13.024	0.993
BMI	-0.033	0.258	-0.543 to 0.478	0.900
Smoking (Yes)	3.124	2.298	-1.425 to 7.674	0.177
Alcohol drinking (Yes)	-3.614	3.059	-9.670 to 2.442	0.240
Tea drinking	-0.092	1.409	-2.882 to 2.698	0.948
ZnO and TiO_2 NPs [b]	0.486	0.237	0.017 to 0.954	0.042

[a] Adjusted for BMI, smoking, alcohol drinking and tea drinking habits; [b] log-transformations of exposure index was used.
